# *StMADS11* Subfamily Gene *PfMADS16* From *Polypogon fugax* Regulates Early Flowering and Seed Development

**DOI:** 10.3389/fpls.2020.00525

**Published:** 2020-05-08

**Authors:** Feng-Yan Zhou, Qin Yu, Yong Zhang, Chuan-Chun Yao, Yun-Jing Han

**Affiliations:** ^1^Institute of Plant Protection and Agro-Products Safety, Anhui Academy of Agricultural Sciences, Hefei, China; ^2^Australian Herbicide Resistance Initiative, School of Agriculture and Environment, University of Western Australia, Perth, WA, Australia

**Keywords:** *Polypogon fugax*, herbicide resistance, flowering regulation, MADS family, *Arabidopsis thaliana*

## Abstract

The evolution of herbicide resistance in weedy plants leads to various adaptation traits including flowering time and seed germination. In our previous studies, we found an association of the early flowering phenotype with the ACCase inhibitor herbicide resistance genotype in a population of *Polypogon fugax*. MADS-box transcription factors are known to play pivotal roles in regulating plant flowering time. In this study, a *SHORT VEGETATIVE PHASE* (*SVP*)*-*like gene, belonging to the *StMADS11* subfamily in the MADS-box family, was cloned from the early flowering *P. fugax* population (referred to as *PfMADS16*) and resistant to the herbicide clodinafop- propargyl. Overexpression of the *SVP*-like gene *PfMADS16* in *Arabidopsis thaliana* resulted in early flowering and seed abortion. This is consistent with the phenotypic characters of resistant *P. fugax* plants, but contrary to the conventional role of *SVP-like* genes that usually suppress flowering. In addition, down regulation of the seed formation gene *AtKTN1* in flowers of *PfMADS16* transgenic Arabidopsis plants indicates that *PfMADS16* may be indirectly associated with seed viability. Furthermore, one protein (PfMADS2) from the *APETALA1* (*AP1*) subfamily interacting with *PfMADS16* in *P. fugax* was identified with relevance to flowering time regulation. These results suggest that the *PfMADS16* gene is an early flowering regulation gene associated with seed formation and viability in resistant *P. fugax* population. Our study provides potential application of *PfMADS16* for integrated weed management (such as genetic-based weed control strategies) aiming to reduce the soil weed seedbank.

## Introduction

Herbicide weed control is the dominant and most intensive selective force imposed in modern agriculture, resulting in widespread evolution of herbicide resistance in many weed species worldwide (Heap, 2020). Herbicide-resistant plants exhibit changes in leaf canopy shape (Bravo et al., 2017), plant size and growth rate (VanEtten et al., 2016; Bravo et al., 2017), flowering time and seed germination rate (Wang et al., 2010) compared to susceptible counterparts. Stress-induced flowering is the third category of flowering response, in addition to photoperiodic flowering and vernalization (Takeno, 2016). In recent years, there have been increasing reports of early flowering in herbicide-resistant populations (Zhu et al., 2013; Tang et al., 2015). For example, a *Hordeum glaucum* biotype resistant to ACCase-inhibiting herbicides flowered earlier than the susceptible biotype in the field and exhibited reduced seed production in competition with *Lens culinaris* (Shergill et al., 2016). A glyphosate-resistant population of *Conyza bonariensis* flowered 28 days earlier and had higher seed germination and production than the susceptible population (Kaspary et al., 2017).

It is well established that flowering is controlled by multiple regulatory genes and pathways and is influenced by environmental conditions (Mouradov et al., 2002; Srikanth and Schmid, 2011). Plant MADS-box transcription factors are key regulators of many developmental processes. MADS-box genes can be classified into 14 clades: StMADS11, AGL17, AGL12, TM3, FLOWERING LOCUS C (*FLC*), AGL6, AGL2, SQUA, AG, TM8, OsMADS32, DEF/GLO, GGM13, and AGL15 (Gramzow and Theissen, 2013; Chen et al., 2017). The *StMADS11* subfamily (isolated from *Solanum tuberosum* L.) of MADS-box genes is predominantly expressed in vegetative tissues and plays important roles in vegetative development and flower transitioning in diverse plant species (Carmona et al., 1998; Cohen et al., 2012; Cooke et al., 2012). The *StMADS11* subfamily contains *AGAMOUS-Like* 24 (*AGL24*) and *SHORT VEGETATIVE PHASE* (*SVP*) genes (Becker and Theissen, 2003) that are involved in the regulation of inflorescence structure and floral organ building (Smith et al., 2011; Liu et al., 2013). *SVP* usually acts as a repressor of the transition to flowering (Hartmann et al., 2000), while *AGL24* acts as a promoter of this process in Arabidopsis (Michaels et al., 2003). Genes of the *StMADS11* family have been identified in various plant species with variable functions. Among the three *StMADS11*-like genes (*OsMADS22*, *OsMADS47*, and *OsMADS55*) in rice, only *OsMADS55* controls flowering time when expressed in Arabidopsis (Fornara et al., 2008; Lee et al., 2012). Overexpression of kiwifruit *SVP3* in *Actinidia eriantha* affects reproductive development, causing abnormal flower, fruit and seed development (Wu et al., 2014). Although extensive research has been carried out on crop plants, little is currently known about flowering time gene regulation in weedy plant species in relation to herbicide resistance.

In our previous research we found that clodinafop-propargyl resistance in a *Polypogon fugax* population (R) is associated with an early flowering phenotype relative to the susceptible population (S) (Tang et al., 2015). Transcriptome analysis identified a flowering-related contig (CL4600.contig2, thereafter named *PfMADS16*) belonging to the *StMADS11*-subfamily of the MADS-box gene family that had significantly higher expression at the flowering stage in R vs. S *P. fugax* (Zhou et al., 2017). We hypothesized that this gene may be involved in early flowering in *P. fugax*, despite its established role in the suppression of flowering in Arabidopsis (Li et al., 2008). In the current study, we transformed *A. thaliana* with *PfMADS16*, and found it caused early flowering. More importantly, overexpression of the *PfMADS16* gene in Arabidopsis resulted in some pods with aborted seeds, which is similar to observations in R *P. fugax* plants. Furthermore, one interacting protein (PfMADS2) with high homology to *Lolium temulentum* MADS2 was also highly expressed in R *P. fugax* during the transition from vegetative to reproductive growth. Therefore, *PfMADS16* is likely involved in early flowering and abnormal seed formation in R *P. fugax* population by interacting with *PfMADS2*. To our knowledge, this is the first report revealing a *StMADS11*-like gene *PfMADS16* that is related to both early flowering regulation and seed abortion in herbicide resistant weed species.

## Materials and Methods

### Plant Material and Growth Conditions

Seeds of the clodinafop-propargyl resistant population of *P. fugax* (referred to as R) were collected from Qingsheng County (29° 54′ 1″ N, 103° 48′ 57″ E), Sichuan Province, China. An herbicide susceptible *P. fugax* population (S) was sampled from a non-cultivated area in Xichang City in Sichuan (27° 50′ 56″ N, 102° 15′ 53″ E). The R and S *P. fugax* populations were characterized in our previous studies (Tang et al., 2014, 2015). In this current study, seeds of the fourth generation of the R and S populations were used that were generated by self-crossing in isolation. The seedlings were transplanted into individual 1 L pots containing potting medium (1:1:1:2 vegetable garden soil/compost/peat/dolomite) after germination. Plants were grown in a glasshouse under natural sunlight with average temperatures of 20/10°C (day/night).

*Arabidopsis thaliana* (L.) Heynh Columbia (*Col*) was purchased from the SALK collection^1^ for transgenic manipulation. Arabidopsis seedlings were transplanted into individual 0.25 L pots containing potting medium (1:1:4 vermiculite/perlite/sphagnum) and were grown at 20°C at 100 μmol m^–2^ s^–1^ photo density under cool white fluorescent light with a photoperiod of 16/8 h (long day condition, LD) or 8/16 h, light/dark (short day, SD).

### Gene Cloning, Molecular Characterization and Phylogenetic Analysis of *PfMads16* cDna From *P. fugax*

In our previous study, we found that the CL4600.Contig2 showed significantly higher expression in R vs. S populations at the reproductive growth stage (Zhou et al., 2017). The open reading frame (ORF) of the contig sequence was predicted using the ORF finder software^2^, and the full-length ORF was cloned using the primers listed in Supplementary Table S1. Isolation of total RNA from R and S *P. fugax* populations and reverse transcription were performed using commercial kits [Takara Biomedical Technology (Beijing) Co., Ltd.]. The amplified full-length cDNA fragment was then ligated into the pMD18-T vector, and confirmed as a homologous gene of the *StMADS11* subfamily based on sequence homology, namely, *PfMADS16*.

Phylogenetic analysis was implemented using the MEGA software version 5.0, and the robustness of the inferred phylogeny was validated by including 1,000 bootstrap replicates.

### Plasmid Construction and Arabidopsis Transformation

The plasmid vectors pCAMBIA2300 and pCAMBIA1303 were digested by *Hin*dIII and *Eco*RI, respectively. The (*CaMV*) 35S promoter of pCAMBIA2300 (1,008 bp) and the large skeleton of pCAMBIA1303 were recovered and purified. Then, T4 DNA ligase (TaKaRa) was used to connect the two parts, and a new two-element expression vector *pCAMBIA1303-35S:35ST* (referred to as the empty plasmid control, Mock) including the 35S promoter was obtained.

The full-length ORF of the *PfMADS16* gene was ligated into the control vector *pCAMBIA1303-35S:35ST* to generate the plasmid *pCAMBIA1303-35S-35ST: PfMADS16* (Supplementary Figure S1A). The plasmid was transferred into WT Arabidopsis plants (*Col*) using the floral dipping method mediated by *Agrobacterium tumefaciens* strain GV3101. All transgenic Arabidopsis seeds (T_0_) were screened on 1/2 MS solid medium containing 50 mg-L^–1^ hygromycin. After germination, T1 transgenic lines (*n* = 40) were verified by PCR amplification of the hygromycin gene and histochemical localization of the GUS gene (Supplementary Figure S1B). Introduction of the target gene (*PfMADS16*) was verified by PCR in positive T2 generation plants (*n* = 36), which all showed an early flowering phenotype compared to the WT. Therefore, the four T2 lines differing slightly in flowering time were used to produce four T3 lines for the following experiments.

### Flowering Time and Seed Production in Transgenic Arabidopsis and Seed Activity Measurement in *P. fugax*

To measure flowering time, untransformed WT, empty plasmid control (Mock) and transgenic plants (*PfMADS16*) were placed on MS agar medium after being surface sterilized with 10% hypochlorite, and were stratified at 4°C for 48 h before being placed at room temperature (22°C). Ten-day-old seedlings (four leaves) were transferred to growth medium (1:1:4 of vermiculite, perlite and sphagnum) and grown under LD (16 h light) or SD (8 h light) conditions.

The flowering times of 20 plants from four T3 transgenic lines were recorded from the day of transplanting until the first flower bloomed. Rosette leaves were counted at the peduncle (2 cm) stage, and the above-ground plant height and total pod numbers were determined on day 55 after transplanting. Seeds were collected on day 62 after transplanting and weighed after drying at 37°C for 24 h.

Seed viability in R and S *P. fuax* was tested using the TTC (triphenyltetrazolium chloride) method. The seeds were soaked in warm water (30°C) for 6 h, cut in half and placed in 0.2% TTC in darkness at 30°C for 24 h. After washing three times with distilled water, color development in the seed endosperm was immediately examined under a stereomicroscope.

### Yeast Two-Hybrid Assay in R *P. fugax*

The yeast two-hybrid library of R *P. fugax* (cloned into the prey vector pGADT7) was constructed by selecting three *P. fugax* R plants randomly at the early flowering stage and using the Matchmaker^®^ Gold Yeast Two-Hybrid System (Clontech) according to the manufacturer’s instructions. Full-length *PfMADS16* was cloned into vector pGBKT7 (bait vector) and then transformed into the yeast strain Y2HGold using the Yeastmaker^TM^ Yeast Transformation System 2 (Clontech).

The constructed R *P. fugax* yeast two-hybrid library was used to screen the interaction proteins of *PfMADS16* according to the manufacturer’s instructions (Matchmaker^®^ Gold Yeast Two-Hybrid System). The primers used for pGBKT7 vector construction are listed in Supplementary Table S1. To confirm protein interactions, the screened prey and bait vectors were validated by one-to-one interaction hybridization.

### Gene Expression Analysis in Arabidopsis and *P. fugax*

To analyze the expression pattern of *PfMADS16* in different tissues of transgenic Arabidopsis plants, plants from the two T3 transgenic lines (35S:*PfMADS16* 1^#^ and 2^#^) were used. Leaf and flower samples were collected at the seedling (6–8 leaves) and flowering (full open) stages, and root, stem and pod samples were collected at the podding stage. Harvested samples were snap frozen in liquid nitrogen for later use.

The whole above-ground parts of *PfMADS16* transgenic (35S:*PfMADS16* 1^#^ and 2^#^) and WT plants were collected at their respective flowering stage for analysis of the expression patterns of four Arabidopsis endogenous genes *FLOWERING LOCUS T* (*FT*), *SUPPRESSOR OF CONSTANS OVEREXPRESSION1* (*SOC1*), *FLOWERING LOCU C* (*FLC*), and *LEAFY* (*LFY*), relevant to flowering regulation, and one gene (*AtKTN1)* relevant to seed formation regulation.

To compare the expression pattern of *PfMADS16* and its interacting protein (PfMADS2) in R and S *P. fugax* plants at each developmental stage, tissue samples of the R and S *P. fugax* plants (*n* = 3) were collected at the seedling and tillering stages, and the samples collected at the early flowering stage of R plants corresponded to the heading stage of the S plants.

Total RNA was extracted using the SGTriEx Total RNA extract Kit (SinoGene), and DNA contamination was removed by RNase-free DNase (Fermentas). The DNA-free RNA was then used for reverse transcription using a Thermo First cDNA Synthesis Kit (SinoGene). The *ACTIN2* and *EF1* genes were used for the normalization of Arabidopsis and *P. fugax* samples, respectively. The primer sequences used for RT-qPCR are provided in Supplementary Table S1. The qPCR was conducted for up to 40 cycles using the following thermal profile: denaturation at 95°C for 15 s, annealing at 55°C for 15 s and extension at 72°C for 45 s. The RT-qPCR results were expressed as means ± SE of three biological replicates each performed in triplicate. Gene expression was calculated as 2^–ΔΔ*Ct*^.

## Results

### Sequence Analysis of *PfMADS16* cDNA From *P. fugax*

The *PfMADS16* coding sequence (651 bp, GenBank accession MN101552) was the same in R and S *P. fugax* plants, encoding a 216-amino acid protein with 86, 84, and 82% identity to known SVP like genes *Festuca arundinacea* VRT2 (ADK55060.1), *Festuca pratensis* MADS-box transcription factor MADS16 (ADW23676.1) (Ergon et al., 2013), and *Lolium perenne* MADS16 (AAZ17551.1), respectively. However, *PfMADS16* only showed 50, 45, and 32% protein identity with *SVP*, *AGL24*, and *SOC1* in Arabidopsis, respectively.

Phylogenetic analysis of *PfMADS16* and SVP/StMADS11-like genes from plant species showed that *PfMADS16* clustered closely to the SVP-like genes of *F. pratensis* MADS16 (ADW23676.1) and *L. perenne* MADS16 (AAZ17551.1), and rice OsMADS22, OsMADS55 and OsMADS47 (Sentoku et al., 2005), *Dimocarpus longan* DlSVP, Arabidopsis SVP, *Aquilegia formosa* AfSVP.2, and *Magnolia praecocissima* MpMADS1. They all clustered in the SVP/StMADS11-like group (Supplementary Figure S2A). This shows that *PfMADS16* most likely belongs to the SVP group, which includes the SVP homologs (NP_179840.2) from Arabidopsis. This group is distinct from *Arabidopsis* AGL24 and SOC1 proteins (Supplementary Figure S2A). Sequence alignments revealed that *PfMADS16* has a conserved MADS-box domain and SVP motif (Supplementary Figure S2B). Thus, *PfMADS16* appears to be an SVP/StMADS11-like transcription factor.

### Overexpression of *PfMADS16* in Arabidopsis Induces Early and Prolonged Flowering, and Abnormal Seed Development

**FIGURE 1:**
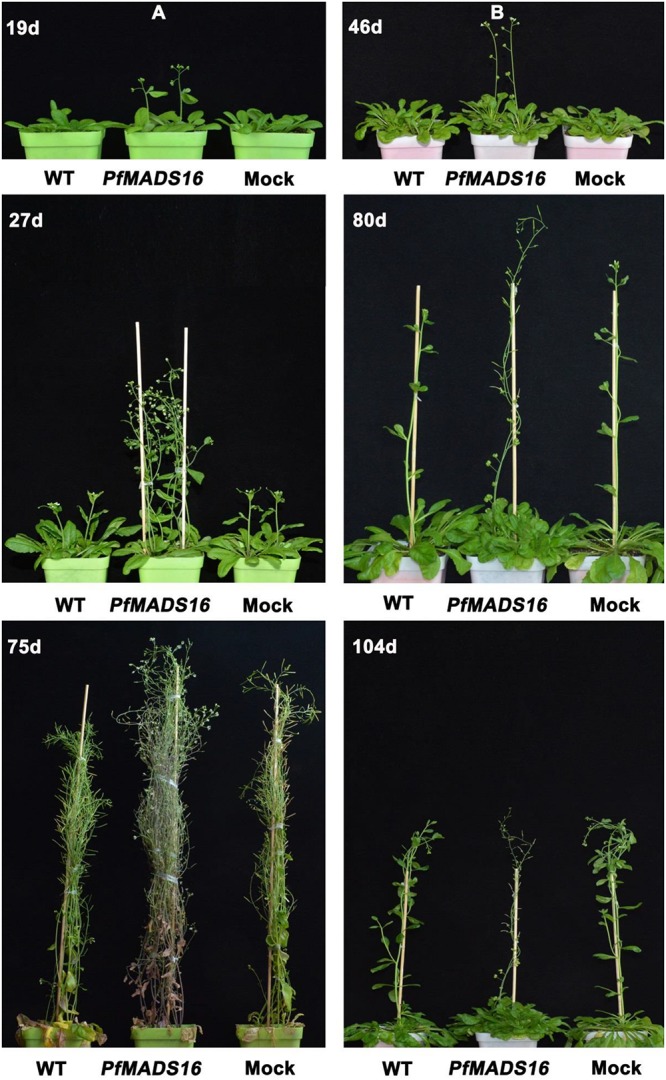
Flowering phenotypes of representative *PfMADS16* transgenic *Arabidopsis thaliana* plants under long day (LD) **(A)** and short day (SD) conditions **(B)** in comparison to untransformed WT and Mock transgenic plants. Photos were taken 19, 27, and 75 days after transplanting under the LD conditions, and 46, 80, and 104 days after transplanting under SD conditions.

Arabidopsis *PfMADS16* T3 transgenic plants flowered 7–9 days earlier and produced 3–4 fewer rosette leaves than WT and Mock plants under the LD conditions (Table 1 and Figure 1A). Under the SD conditions. *PfMADS16* transgenic plants flowered ~25–42 days earlier but produced 9–13 more rosette leaves than control plants (Table 1 and Figure 1B). The flowering period of *PfMADS16* transgenic plants lasted significantly longer than the controls under the LD conditions, and while all the control plants produced pods, the *PfMADS16* transgenic plants were still producing new flowers (Figure 1A, 75 days).

**TABLE 1:** Changes in growth and reproduction of *Arabidopsis thaliana* overexpressing the *PfMADS16* gene under long day (LD) and short day (SD) conditions.

**Treatment**	**No. rosette leaves**	**Flower time (day)**	**Pod numbers**	**Plant height (cm)**	**Seed yield (g plant^–1^)**
**Long day condition (LD)**					
WT	13.85 ± 0.20^b^	27.90 ± 0.33^b^	280 ± 6^b^	39.26 ± 0.53^b^	0.1405 ± 0.0031^b^
Mock	13.50 ± 0.24^b^	28.10 ± 0.28^b^	279 ± 6^b^	40.27 ± 0.44^b^	0.1382 ± 0.0018^b^
*PfMADS16*	10.65 ± 0.22^a^	19.70 ± 0.18^a^	394 ± 9^a^	50.36 ± 0.88^a^	0.0662 ± 0.0039^a^
**Short day condition (SD)**					
WT	48.10 ± 1.51^b^	80.80 ± 1.39^b^	61 ± 2^b^	48.53 ± 0.38^a^	0.0319 ± 0.0012^b^
Mock	44.65 ± 1.36^b^	83.70 ± 2.01^b^	64 ± 2^b^	50.43 ± 0.63^a^	0.0329 ± 0.0010^b^
*PfMADS16*	59.10 ± 0.93^a^	47.00 ± 0.42^a^	73 ± 1^a^	49.23 ± 0.73^a^	0.0191 ± 0.0004^a^

*Data was mean ± SE of 20 Arabidopsis plants from four T3 lines. Different letters in a column indicate a significant difference based on Ducan’s multiple comparison test, p < 0.01.*

**FIGURE 2:**
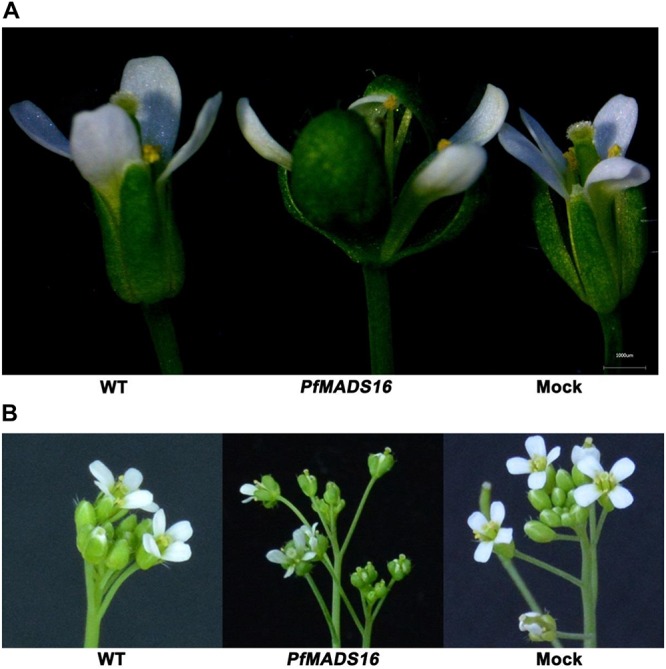
Representative phenotype changes in flowers of *PfMADS16* transgenic Arabidopsis plants in comparison to WT and Mock plants. **(A)** Morphological differences of a single flower. **(B)** Morphological differences of the inflorescence. Flower phenotype changers among transgenic lines are similar.

**FIGURE 3:**
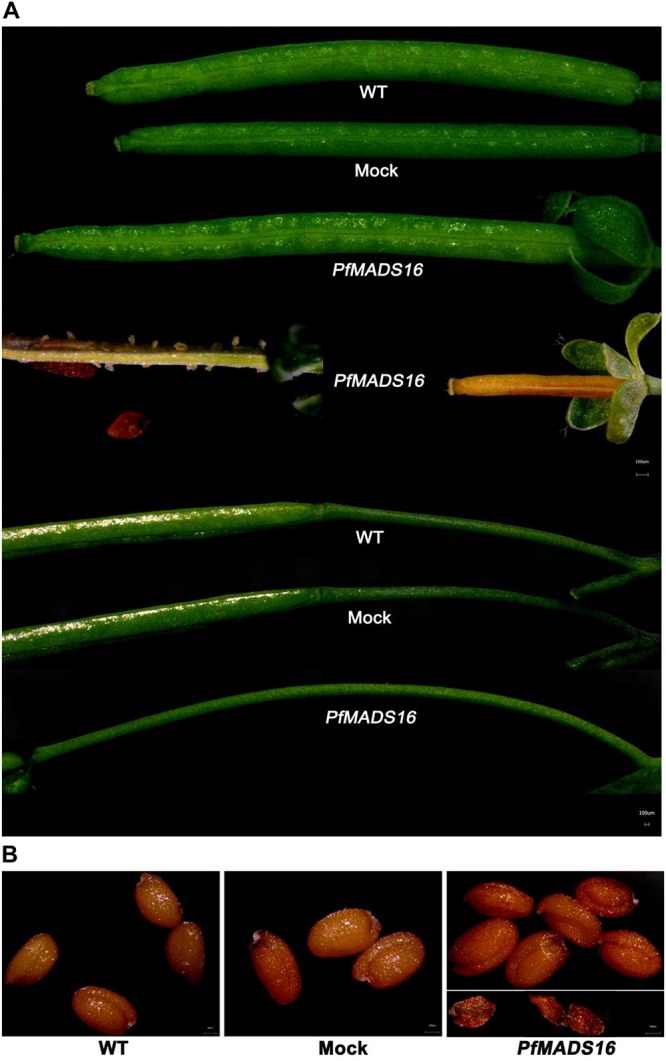
Phenotypic abnormalities in podding of representative *PfMADS16* transgenic Arabidopsis plants as compared to WT and Mock plants. **(A)** *PfMADS16* transgenic plants exhibiting different pod types (long and short), sepals that do not fall off at maturity, and elongated pedicels. **(B)** *PfMADS16* transgenic plants with aborted seeds. Similar results were observed among T3 transgenic lines.

**FIGURE 4:**
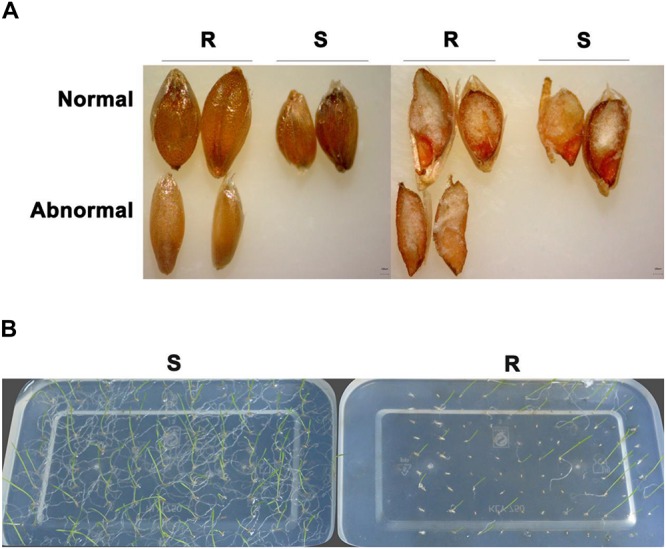
Comparison of seed vigor and the germination rate between resistant (R) and susceptible (S) of *Polypogon fugax*. **(A)** Seed appearance (left) and the seed embryo activity test by the TTC method (right) in R and S plants. **(B)** Seed germination (23 day) of R and S plants on 0.6% agar at 15°C.

In contrast to control plants, the flowering pattern of *PfMADS16* transgenic plants became choripetalous (Figures 2A,B), with sepals wider and rounder than normal that did not fall off even when the pods were mature; in addition, the length of the peduncle was markedly increased (Figure 3A). Interestingly, single *PfMADS16* transgenic plants produced two types of pods: normal ones similar to WT, and abnormal ones (more than half of the total pods) with aborted seeds (Figure 3B). This was similar to the R *P. fugax* plants that also had two types of seeds: normal ones similar to the S *P. fugax* seeds, and the abnormal ones with a low endosperm content and lack of seed viability (Figure 4A). There was a significant difference in the 100-grain weight (7.7 vs. 9.4 mg, *p* < 0.05) between R and S *P. fugax*, and R seeds had a lower percentage germination rate than the S seeds (Figure 4B). Although *PfMADS16* transgenic Arabidopsis plants were taller and had more total pods than WT, seed production was significantly lower than the control plants (Table 1). Thus, overexpression of *PfMADS16* in Arabidopsis resulted in a phenotype with early-flowering and seed abortion.

### Expression Pattern of *PfMADS16* and Endogenous Genes Involved in Flowering and Seed Formation in Transgenic Arabidopsis

**FIGURE 5:**
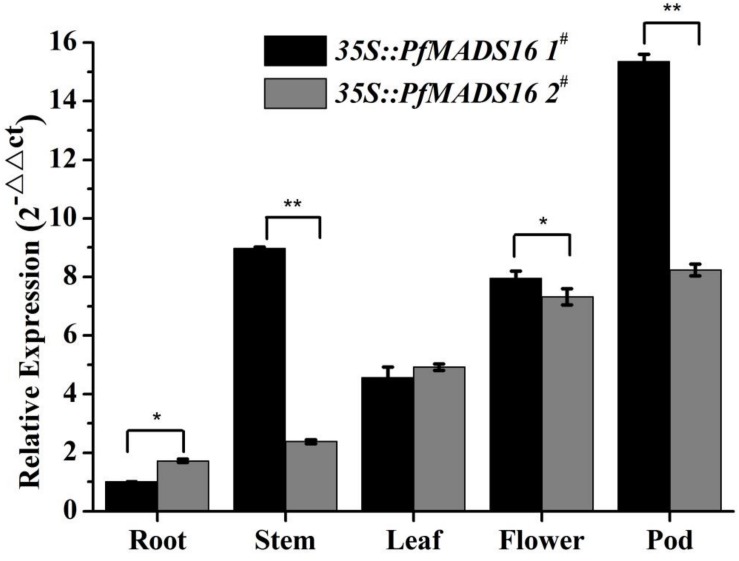
RT-qPCR analysis of *PfMADS16* gene expression in different tissues of plants from two T3 transgenic Arabidopsis lines. Error bars represent standard error calculated from three biological replicates. An actin homologous gene (*ACTIN2*) of Arabidopsis was used as an internal control. *indicates significant difference, *p* < 0.05; **indicates significant difference, *p* < 0.01.

The expression of *PfMADS16* in different tissues of transgenic Arabidopsis plants was analyzed by RT-qPCR. Results from the two T3 lines are similar showing that *PfMADS16* were expressed in almost all of the tissues examined, with relatively higher transcript levels in the pods, flowers and leaves than in the roots (Figure 5). Therefore, a high level of *PfMADS16* expression in flowers and pods might contribute to flowering and pod development.

**FIGURE 6:**
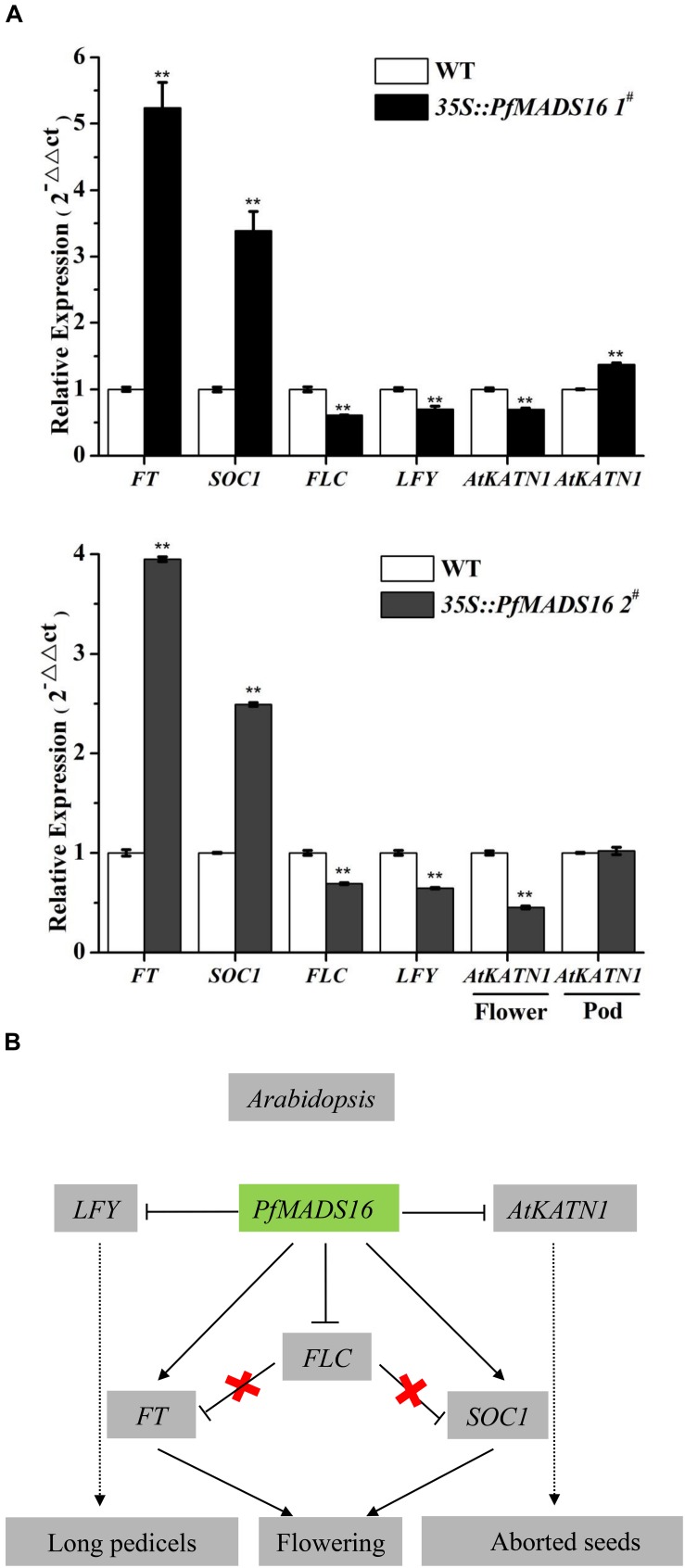
Overexpression of *PfMADS16* promotes flowering in Arabidopsis. **(A)** RT-qPCR analysis of *PfMADS16* and the four endogenous flowering-related genes (*FT*, *SOC1*, *FLC*, *LFY*) in whole above-ground material of the two transgenic Arabidopsis lines and WT at the early flowering stage, analysis of *AtTKN1* in flowers and pods of transgenic and WT plants. The *ACTIN2* gene was used as an internal control. ** indicates significant difference, *p* < 0.01. **(B)** Proposed model of flowering induction in *PfMADS16* transgenic Arabidopsis plants. *FLC*, Flowering locus C; *SOC1*, Suppressor of over-expression of CO1; *FT*, Flowering locus T; *LFY*, LEAFY; Arrow, enhanced expression; Horizontal line, inhibited expression; Cross, interrupted expression; Dotted line, indirect promotion.

The expressions of *FT* and *SOC1* were also higher, while those of *FLC* and *LFY* were lower in above-ground material of both lines of *PfMADS16* transgenic Arabidopsis plants compared to the controls (Figure 6A). In addition, the expression of *AtKTN1* was significantly lower in flowers, and higher in pods of 35S:*PfMADS16* 1^#^ (but not in the 35S:*PfMADS16* 2^#^) plants compared to the WT control (Figure 6A).

### Expression Pattern of *PfMADS16* and the Interacting Protein in *P. fugax*

**FIGURE 7:**
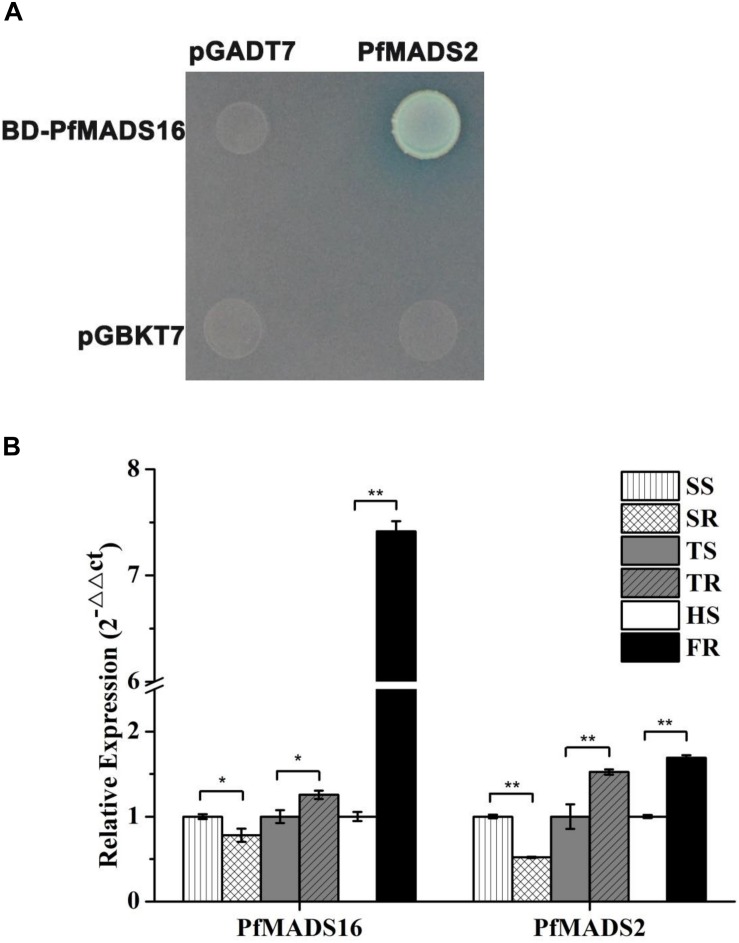
The yeast two-hybrid and expression assays. **(A)** Protein interaction was indicated by the ability of yeast cells to grow on synthetic dropout medium lacking Leu/Trp/His/Ade. Genes for PfMADS16 and the interaction protein (PfMADS2) were cloned into pGBKT7 (shown as BD) and pGADT7 (shown as AD), respectively. **(B)** RT-qPCR analysis of *PfMADS16* and *PfMADS2* in the resistant population of *P. fugax*. * indicates significant difference, *p* < 0.05; ** indicates significant difference, *p* < 0.01. SR, Resistant plants at the seedling stage; SS, Susceptible plants at the seedling stage; TR, Resistant plants at the tillering stage; TS, Susceptible plants at the tillering stage; FR, Resistant plants at the flowering stage; HS, Susceptible plants at the heading stage.

One MADS family protein (named PfMADS2) that interacted with *PfMADS16* in R *P. fugax* was identified by the yeast two-hybrid analysis and was validated (Figure 7A). The protein showed 94.7, 94.3, and 90.2% sequence identity to *L. perenne* MADS-box protein 2 (MADS2) (AAO45874.1), *L. temulentum* MADS2 (AF035379. 1), and *Aegilops tauschii* subsp. tauschii MADS-box transcription factor 15-like (XP_020149442.1), respectively (Supplementary Table S2).

The expression patterns of *PfMADS16* and the interacting protein (*PfMADS2*) were compared at the seedling and tillering stages between R and S plants. In addition, the expression patterns of these genes were also compared at the early flowering stage of the R plants corresponding to the heading stage of the S plants. Results showed the expression of *PfMADS16* was lower at the seedling stage, but higher at the tillering stage in the R compared with the S plants (Figure 7B). The expression level of *PfMADS16* was 7.4-fold higher in the early flowering stage of R than that of S (while S still at the heading stage), which is consistent with the expression pattern of the interacting protein PfMADS2 (Figure 7B). These results suggest that both *PfMADS16* and *PfMADS2* may be associated with early flowering in the R population.

## Discussion

Through Arabidopsis genetic transformation, the current study found that *PfMADS16* is involved in the early flowering and seed formation of *P. fugax*. An unexpected finding is that the *PfMADS16* gene has the *SVP* motif and high identity to known SVP-like genes (Supplementary Figure S2A) but does not function as a normal *SVP* gene. When expressed in Arabidopsis, the *SVP*-like gene *PfMADS16* promoted early flowering, which is in contrast to the results that overexpression of *A. thaliana SVP* led to a late-flowering phenotype (Li et al., 2008). There is a possibility of differential regulation and function of the *SVP-like* genes among plant species. For example, although highly similar to the *PfMADS16* gene, the *FPMADS16* gene has no known function in vernalization-induced flowering in *F. pratensis* (Ergon et al., 2013). Of the four *SVP* homologous genes in kiwifruit, only *SVP1* and *SVP3* were related to the transition to flowering, whereas the other two had distinct roles during bud dormancy (Wu et al., 2012; Voogd et al., 2015). Constitutive expression of *35S:EsSVP* in *Petunia* W115 had little or no effect on flowering time, but clearly affected flower development (Li et al., 2016). In the current study, transgenic Arabidopsis plants expressing *P. fugax PfMADS16* gene showed a mild defect in floral phenotype, with choripetalous petals surrounded by four large leaf-like sepals (Figure 2A), similar to the phenotypes of transgenic Arabidopsis expressing kiwifruit *SVP1* and rice *OsMADS55* (Lee et al., 2012; Wu et al., 2012). In addition, like the phenotypes of transgenic Arabidopsis expressing rice *OsMADS22* and *OsMADS47* (Fornara et al., 2008), the *PfMADS16* transgenic plants had leaf-like sepals that were retained even after fertilization (Figure 3B).

In Arabidopsis, *FLC* is a key gene acting at the convergence point of the vernalization and autonomous pathways and inhibits flowering by suppressing *FT* expression (Komiya et al., 2008). *FLC* and *SVP* form a repressor complex that suppresses the expression of both *SOC1* and *FT* (Tao et al., 2012). However, endogenous *SOC1* and *FT* expression were significantly increased in *PfMADS16* transgenic Arabidopsis plants, and the expression of *FLC* was significantly decreased (Figure 6A). These results indicate that ectopic expression of *PfMADS16* in Arabidopsis may regulate flowering by promoting the expression of *FT* and *SOC1*, causing co-suppression of related repressors such as *FLC*. The *LFY* expression level is an important determinant of flower initiation that negatively controls epidermal cell elongation and stomatal numbers in the pedicel (Yamaguchi et al., 2012). This is consistent with the decreased *LFY* expression (Figure 6A) that led to pedicel prolongation in *PfMADS16* transgenic Arabidopsis plants (Figure 3A). It was noticed that *PfMADS16* transgenic plants flowered earlier but had more rosette leaves than the controls under the SD condition (Table 1 and Figure 1B). Usually, the shorter the number of days prior to bolting the fewer the number of rosette leaves, although some exceptions exist (e.g., Möller-Steinbach et al., 2010). Overexpression of *PfMADS16* may affect other physiological regulation mechanisms in Arabidopsis.

The most interesting finding in the current study is that the *PfMADS16* gene regulates not only early flowering but also seed formation. KATANIN 1 is a microtubule severing protein, and phenotypical abnormalities in embryogenesis and seed formation of KATANIN 1 mutants can be rescued by complementation with the *AtKTN1* gene (Luptovčiak et al., 2017). In *PfMADS16* transgenic Arabidopsis plants, we estimated that nearly half of the pods had aborted seeds, and the expression of *AtKTN1* was lower in flowers but higher in pods compared to the controls (Figure 6A). It is possible that overexpression of *PfMADS16* suppresses *AtKTN1* expression in flowers, causing abnormal seed setting and development in *PfMADS16* transgenic Arabidopsis plants. In contrast, overexpression of *PfMADS16* promotes *AtKTN1* expression in pods, resulting in higher pod numbers than in controls by unknown mechanisms (Table 1). These results indicate that the *PfMADS16* gene might be a flowering time promoter for efficient expression of other flowering time regulating genes, causing early flowering and seed abortion in transgenic Arabidopsis plants, as summarized in Figure 6B.

In order to understand the flowering regulation pathway of *PfMADS16* in R *P. fugax*, we identified an interacting protein *PfMADS2* with homology to *L. temulentum* MADS2 (*LtMADS2*) and *L. perenne* MADS2 (*LpMADS2*). In accordance with the present results, previous studies demonstrated that *LtMADS2* transgenic Arabidopsis plants interact with the *AP1* promoter, causing early flowering (Gocal et al., 2001). During vernalization the *LpMADS2* gene (clustered in the AP1 subgroup) alone may be insufficient for initiating floral transition, and other genes might act together with this vernalization induced gene to initiate the floral transition (Petersen et al., 2004). In our study, the interaction protein (*PfMADS2*) was significantly more highly expressed in R than in S *P. fugax* at the tillering and flowering stages (Figure 7B), and thus we assume that *PfMADS16* may act together with *PfMADS2* to initiate early floral transition in R *P. fugax.* In addition, the phenotype of *PfMADS16* transgenic Arabidopsis plants (Figure 3A) is consistent with that of *LtMADS2* transgenic Arabidopsis plants that had abnormally long pedicels and ectopic flowers (Gocal et al., 2001).

Flowering time regulation is a coherent and sophisticated event, involving many genes. Based on the current experiment data, we can infer that the *PfMADS16* gene is among the major genes that are involved in the early flowering regulation network in the R *P. fugax* population. Questions remain as to how early flowering and resulting seed abortion are associated with resistance evolution in weedy plants. Among other possibilities, herbicide stress may trigger flowering gene expression in both S and R plants, and R plants are able to survive due to the presence of resistance alleles (mutant ACCase alleles in the case of *P. fugax*). The early flowering trait may become inheritable due to epigenetic modification of flower genes and co-segregation with resistance traits. This hypothesis can be tested by measuring flowering gene expression after herbicide treatment and by analysis of flowering gene methylation. Indeed, *PfMADS16*, together with other flowering genes, was highly expressed in both R and S *P. fugax* plants 72 h after *clodinafop-propargyl* treatment at the seedling stage as compared to the untreated control in our previous study (see Figure 6A and Supplementary Table S11) (Zhou et al., 2017).

Alternatively, due to standing genetic variations in gene expression in weed populations, plants with higher expression of flowering genes by randomly co-segregate with herbicide resistance traits. Early flowering plants may escape harvest weed control strategies by early pod shedding. Weed control methods such as harvest weed seed control (HWSC) systems that have been developed to reduce weed seed return to soil during crop harvest (Walsh and Powles, 2014) may not be effective at harvesting seeds of early flowering plants (Ashworth et al., 2016). With increased knowledge on genes regulating flowering time, pod shedding and seed formation in weedy plants, it may be possible in the future to genetically manipulate these genes to reduce weed seed production, through the CRISPR-based gene drive system (Neve, 2018) for better weed control strategies.

## Conclusion

This study demonstrates that the *PfMADS16* gene, different from the normal *SVP*-like genes in Arabidopsis, is an early flowering regulation gene, and is associated with seed formation and viability in R *P. fugax* population. These results will help understand flowering regulation mechanisms in herbicide resistant weeds, and are useful for genetic-based weed control strategies aiming to manipulate the weed flowering time and reduce the soil seed bank.

## Data Availability Statement

The datasets generated for this study are available on request to the corresponding author.

## Author Contributions

F-YZ and QY conceived and designed the experiments and wrote and revised the manuscript. F-YZ and C-CY performed the experiments. YZ and Y-JH analyzed the data. F-YZ, QY, YZ, C-CY, and Y-JH read and approved the final manuscript.

## Conflict of Interest

The authors declare that the research was conducted in the absence of any commercial or financial relationships that could be construed as a potential conflict of interest.

## Footnotes

1 http://signal.salk.edu/

2 https://www.ncbi.nlm.nih.gov/orffinder/
