# Supplementary material for: StMADS11 Subfamily Gene PfMADS16 From Polypogon fugax Regulates Early Flowering and Seed Development
Source: Front Plant Sci. 2020 May 8;11:525. doi: 10.3389/fpls.2020.00525 (PMC7225323; doi:10.3389/fpls.2020.00525)
Supplement: Supplementary file 1 [file Data_Sheet_1.docx]

**SUPPLEMENTARY MATERIAL**

**Supplementary Table S1. Primers used in the study**

| **Primer** | **Sequences (5’-3’)** | **Purpose** | |
| --- | --- | --- | --- |
| FK | GGGGTACCATGGCGCGGGAGAGGCGGGAGATAAAG | | Clone full-length cDNA fragment |
| RB | CGGGATCCTCAAATCAACGCTAGTTTCAGGGATATATCAGAACCGTCATCATTG | |  |
| *PfMADS16-*F | CATGGAGGCCGAATTCATGGCGCGGGAGAGGCGGGAGATAC | | Bait vector construction |
| *PfMADS16-*R | GCAGGTCGACGGATCCTCAAATCAACGCTAGTTTCAGGGAT | |  |
| *ACTIN2*-F | TGCTGGATTCTGGTGATGGT | | Reference gene for Arabidopsis |
| *ACTIN2*-R | AATTTCCCGCTCTGCTGTTG | |  |
| *FLC*-F | CTCTACAGCTTCTCCTCCGG | | Analysis *Flowering locus C* gene expression in Arabidopsis |
| *FLC*-R | TCCCACAAGCTTGCTATCCA | |  |
| *SOC1*-F | ATTCGCCAGCTCCAATATGC | | Analysis *Suppressor of over- expression of CO1* gene expression in Arabidopsis |
| *SOC1*-R | TGTTGCAGCTCCTCGATTGA | |  |
| *FT*-F | AAGTCCTAGCAACCCTCACC | | Analysis *Flowering locus T* gene expression in Arabidopsis |
| *FT*-R | CATACACTGTTTGCCTGCCA | |  |
| *LFY*-F | TGTGAACATCGCTTGTCGTC | | Analysis *LEAFY* gene expression in Arabidopsis |
| *LFY*-R | TAATACCGCCAACTAAAGCC | |  |
| *AtKATN1-F* | TTCTCATGTTTGGTCCTCCTG | | Analysis *AtKATN1* gene expression in Arabidopsis |
| *AtKATN1-R* | ATTGTGCTTGGAGCATATGCC | |  |
| *PfMADS16-*F | CTCGTCGTCTTCTCCTCCAC | | Analysis *pfMADS16* expression in  Arabidopsis *and P. fugax* |
| *PfMADS16-*R | TTCCCCAAGTTCCTCACCTC | |  |
| *EF1*-F | GAACCTCCCAGGCTGATTGT | | Reference gene for *P. fugax* |
| *EF1*-R | CAAGAGTGAAAGCAAGAAGAGCA | |  |
| *PfMADS2-F* | GGGAAATTGGTGCCACGAAT | | Analysis *PfMADS2* expression in *P. fugax* |
| *PfMADS2-R* | CTGCAGTGACCTCTCCTTCT | |  |

**Supplementary Table S2. The sequence of interacting protein PfMADS2**

| **Category** | **Length** | **Sequence** |
| --- | --- | --- |
| Nucleotide | 789bp | ATGGGTCGCGGCAAGGTGCAGCTGAAGCGGATAGAGAACAAGATAAACCGTCAGGTGACCTTCTCCAAGCGCCGCAACGGGCTGCTCAAGAAGGCGCACGAGATCTCCGTCCTCTGCGACGCCGAGGTCGCCGTCATCGTCTTCTCCCCGAAAGGGAAGCTCTACGAGTACGCCACTGACTCCAGCATGGACAAAATTCTTGAACGTTATGAGCGCTACTCCTATGCTGAAAAGGCTCTAATTTCAGCTGAATCTGAAAGTGAGGGAAATTGGTGCCACGAATACAGGAAACTGAAGGCGAAGATTGAGACTATACAAAAATGTCACAAGCACCTCATGGGAGAGGATCTGGACTCTCTGAACCTCAAAGAACTCCAACAACTGGAGCAGCAGCTTGAGAGTTCATTGAAACACATCAGATCGAGAAAGAGCCACCTTATGATGGAGTCCATTTCTGAGCTACAGAAGAAGGAGAGGTCACTGCAGGAGGAGAACAAGGCTCTACAGAAGGAACTGGTGGAGAGGCAGAAGTCGGCCAGGCAGCAGCAGCAGCAGCAAGTGCAGTGGGAGCACCAGACCCAAACACAACAAGCCCAAAACCAACCTCAAGCCCAGACGAGCTCATCCTCTTCCTCCTTCATGATGAGGGATCAGCAGGCACATGCTCAACAAAACATCTGTTACCCGCCGGTGACGATGGGCGGTAATGCGGCCGTGGCACCAGGGCAGCAGGGGCAGCTTCGCATCGGAGGCCTGCCACCATGGATGCTGAGCCACCTCAACGCTTGA |
| Amino acid | 261aa | MGRGKVQLKRIENKINRQVTFSKRRNGLLKKAHEISVLCDAEVAVIVFSPKGKLYEYATDSSMDKILERYERYSYAEKALISAESESEGNWCHEYRKLKAKIETIQKCHKHLMGEDLDSLNLKELQQLEQQLESSLKHIRSRKSHLMMESISELQKKERSLQEENKALQKELVERQKSARQQQQQQVQWEHQTQTQQAQNQPQAQTSSSSSSFMMRDQQAHAQQNICYPPVTMGGNAAVAPGQQGQLRIGGLPPWMLSHLNA |

**A**

**
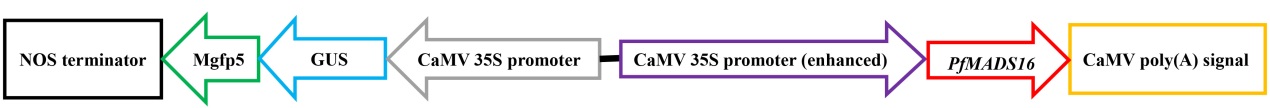
**

**B**

**
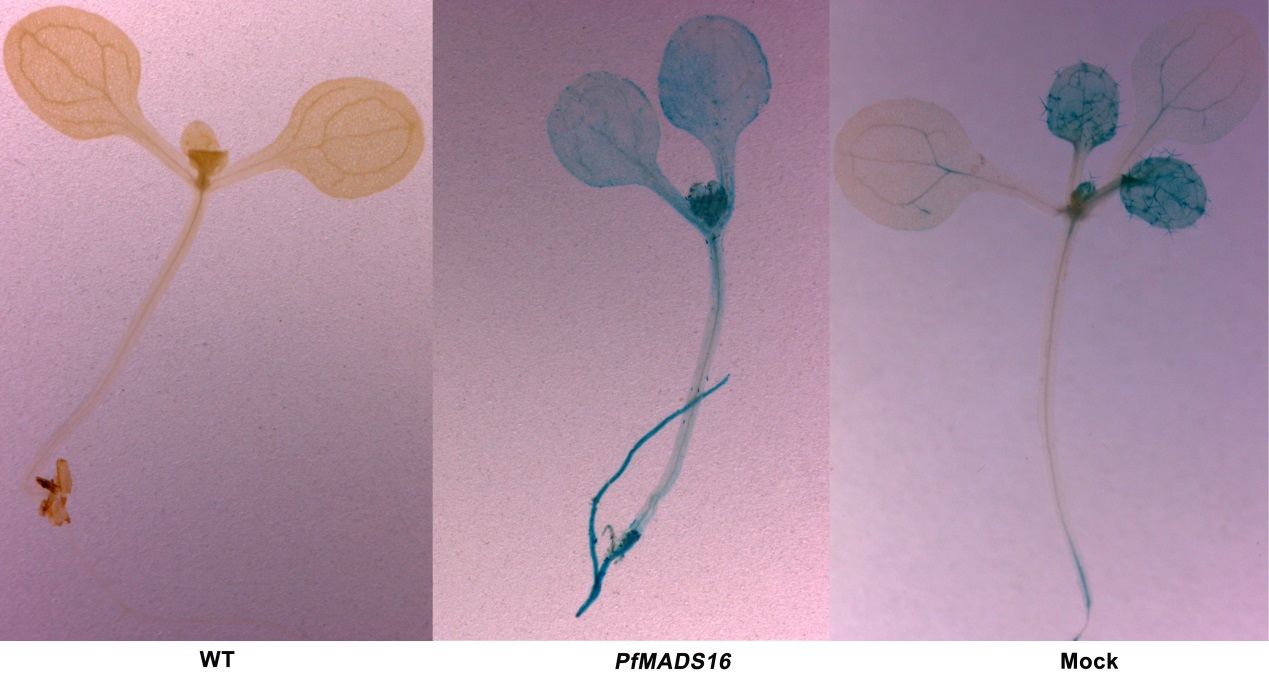
**

**Supplementary Figure S1**. Construction of the *PfMADS16* transgenic vector and expression verification in transgenic plants. (A) Schematic diagram of the T-DNA region of the binary plasmid used in this study; (B) Expression patterns of the *pfMADS16* gene in transgenic Arabidopsis. Plants were grown under long day conditions for 8 d and whole plants were used for GUS staining. WT, wild-type; *PfMADS16*, *PfMADS16* transgenic Arabidopsis; Mock, empty plasmid control.

**A**

**
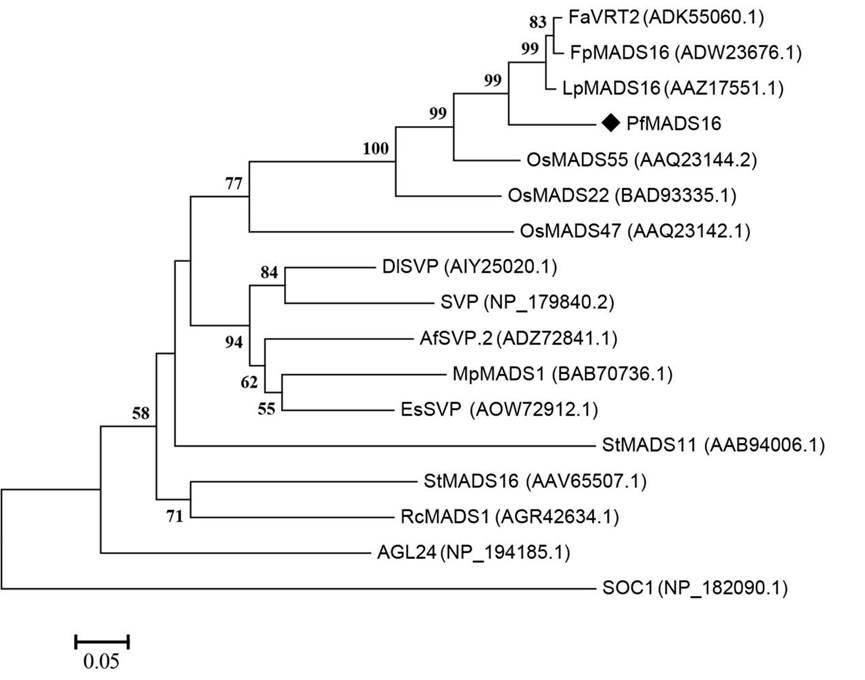
**

**B**

**
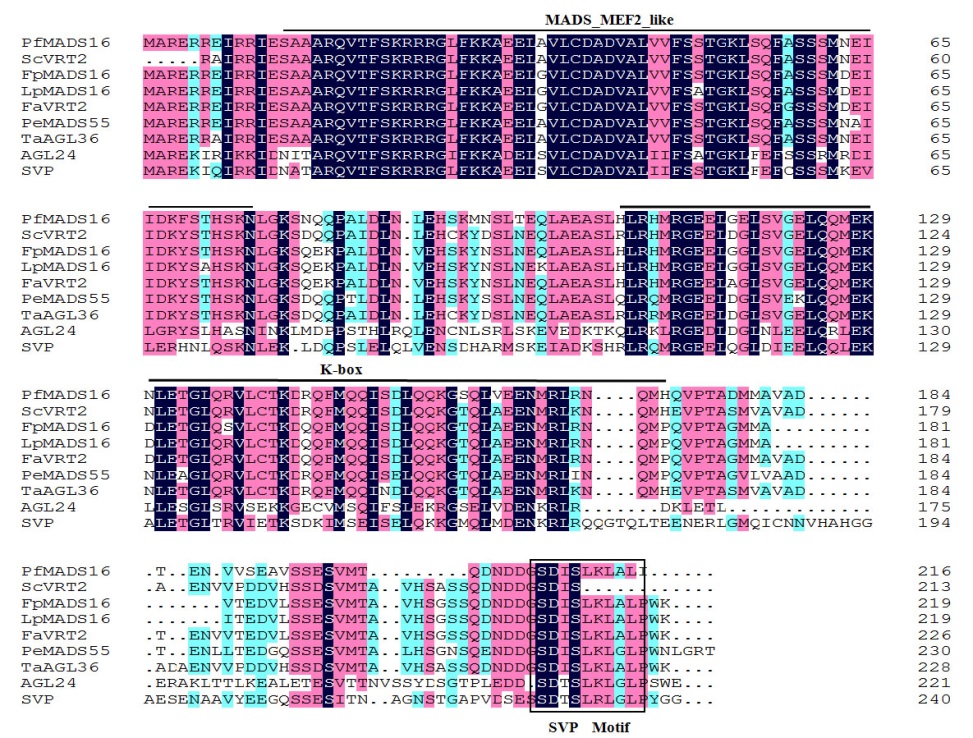
**

**Supplementary Figure S2.** Characterization of the *PfMADS16* gene sequence. (A) Phylogenetic analysis of the *PfMADS16* amino acid sequence; the *SOC1* gene that does not belong to the StMADS11 subfamily was used as an out-group comparison. (B) Deduced amino acid sequence alignment of *PfMADS16* with *ScVRT2* (ADR51708.1), *FpMADS16* (ADW23676.1), *LpMADS16* (AAZ17551.1), *FaVRT2* (ADK55060.1), *PeMADS55* (ASZ79951.1), *TaAGL36* (ABF57936.1), *AGL24* (OAO97217.1) and *SVP* (OAP09056. 1). The MADS-MEF2 domain, K-box domain and SVP motif are indicated.
